# Supplementary material for: Patterns of Elder Caregiving Among Nigerians: An Integrative Review
Source: Int J Environ Res Public Health. 2025 Dec 19;23(1):2. doi: 10.3390/ijerph23010002 (PMC12840742; doi:10.3390/ijerph23010002)
Supplement: Supplementary file 1 [file ijerph-23-00002-s001.zip › ijerph-3897781-supplementary-finalo.pdf]

Supplementary File S1

Comprehensive Search Strategy.

Search terms	Database	Citations
aging or ageing or elderly or older adults or seniors or geriatrics or gerontology) AND (Nigeria or Nigeria, Eastern or Nigeria, Western or Nigeria, Eastern or Nigeria, Southern or Nigeria, Northern or Nigerian, Overseas, Nigerian, abroad) AND (Care or caregiv* or support)	Academic Search Complete (Ebsco)	627
"(aging or ageing or elderly or older adults or seniors or geriatrics or gerontology) AND (Nigeria or Nigeria, Eastern or Nigeria, Western or Nigeria, Eastern or Nigeria, Southern or Nigeria, Northern or Nigerian, Overseas, Nigerian, abroad) AND (Care or caregiv* or support)" OR (MH "Nigeria") OR (MH "Eldercare")	CINAHL (Ebsco)	331
"(aging or ageing or elderly or older adults or seniors or geriatrics or gerontology) AND (Nigeria or Nigeria, Eastern or Nigeria, Western or Nigeria, Eastern or Nigeria, Southern or Nigeria, Northern or Nigerian, Overseas, Nigerian, abroad) AND (Care or caregiv* or support)" OR (MH "Nigeria") OR (MH "Eldercare")	PsychINFO (Ebsco)	532
aging or ageing or elderly or older adults or seniors or geriatrics or gerontology) AND (Nigeria or Nigeria, Eastern or Nigeria, Western or Nigeria, Eastern or Nigeria, Southern or Nigeria, Northern or Nigerian, Overseas, Nigerian, abroad) AND (Care or caregiv* or support)	PubMed (Medline)	612
aging or ageing or elderly or older adults or seniors or geriatrics or gerontology) AND (Nigeria or Nigeria, Eastern or Nigeria, Western or Nigeria, Eastern or Nigeria, Southern or Nigeria, Northern or Nigerian, Overseas, Nigerian, abroad) AND (Care or caregiv* or support)	Medline (OVID)	884
**Total retrieved**		**2,986**

Supplementary File S2

PRISMA Flow Diagram of the Inclusion Process.

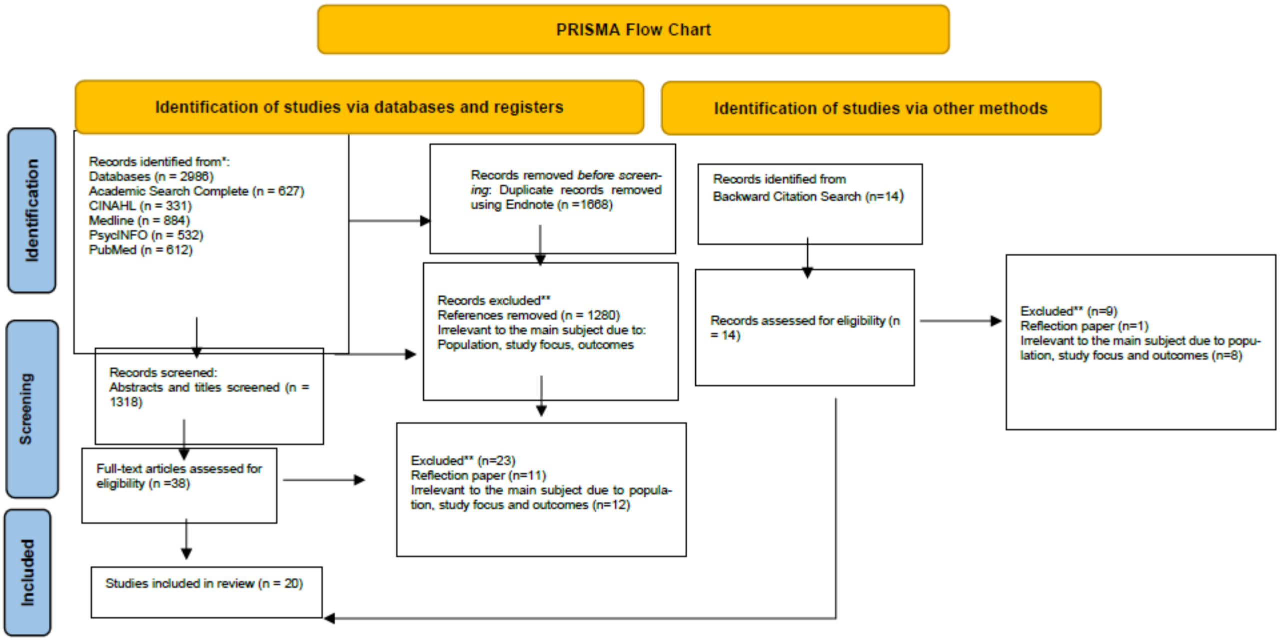

Supplementary File S3

Data Extraction Matrix

Author / Journal / Year	Sample / Population	Objectives	Methodol ogy / Study Design	Data Collection Methods	Cultural Influences	Gender Difference s	Family Dynamics	Economic Factors	Caregiver Strain and Psychosocial Dimensions	Governmen t Policies and Support
Akinrolie et al., Gerontologi cal Social Work (2020)	18 participant s (10 elderly, 8 adult children) in Abuja	To explore the perception of reciprocity between older parents and adult children on intergenerati onal support in Northern Nigeria	Qualitativ e	Interviews	Rapid modernizati on in developing countries had negative effects, according to elderly individuals.		Support was seen as a continuous process throughout life, flowing in multiple directions. Some participants relocated their parents from rural areas, assuming responsibility for their needs and medical expenses. Elderly individuals categorized support into material, monetary, and physical assistance.	Elderly individuals perceived a decline in support received, attributing it to the country's deteriorating economic situation. Monetary support from adult children was emphasized, while emotional support was less emphasized.	Types of support received were influenced by adult children's busy schedules and limited availability.	
Ani & Isiugo-Abani,	444 respondent s aged 65+	To explore the conditions of	Mixed methods	Questionna ires and Interviews	Rapid modernizati on in		Elderly individuals with larger families	Some elderly individuals relied on pensions. 66.4% of respondents aged 65 and above continued		The absence of government

International Journal of Sociology of the Family (2017)	from selected LGAs of Ibadan, Oyo State	the elderly in Ibadan, Nigeria.			developing countries had negative effects, according to elderly individuals.		had the expectation of receiving support in old age.	working to compensate for inadequate care from children. Support received was often inadequate; urban elders were twice as likely to receive adequate care compared to rural elders. Variables such as education, residence, number of children, and income were associated with receiving adequate care.		social security programs led to the expectation of children as primary providers of care.
Animasahun & Chapman, African Health Sciences (2017)	Studies conducted between 2000 and 2015	To examine the driving factors for the psychosocial health challenges among the elderly in Nigeria	Narrative Review			Gender imbalance affects social support, with older women facing more challenges and domestic violence. Women are financially under-resourced and experience more psychosocial health challenges in older age compared to men.	Family provides up to 90% of home care, leading to caregiver stress and health risks. Materialism and abandonment are replacing Nigerian family values, resulting in insecurity and increased abuse or neglect of the elderly. Economic pressures cause migration to urban areas, impacting the traditional extended family structure.	Economic pressures cause family members to migrate to urban areas, impacting the traditional extended family structure.	Caregiver stress and health risks.	Geriatric medical services are not prioritized, leading to lengthy waiting times, low provider-patient ratios, and poor communication.
Ebimgbo et al., Indian Journal of	40 caregivers aged 23+	To gain understanding on	Qualitative	Focus group discussions				Participants spent significant amounts of money on food and drugs for elderly individuals.	Participants experienced stress while	

Gerontology (2019)	living in Nnewi, south-east Nigeria	challenges of caregiving to elderly individuals								caring for elderly individuals. Family caregivers had limited time for themselves and their own families due to the demands of caregiving.	
Ebimgbo & Okoye, Nigerian Journal of Social Sciences (2017)	528 elderly individuals in Nnewi, Southeast, Nigeria	To ascertain the factors that predict availability of social support whenever it is needed by elderly individuals	Quantitative study	Questionnaires			Financial status, attitudes of children, and health status were substantial in predicting the receipt of social support by elderly individuals. Family networks (children) provide the most support, followed by churches, government/NGOs, community, and friends. Attitudes of adult children shape support; better parent–child relationships				Low financial background reduced likelihood of receiving support; 64.6% reported support inadequate or unsatisfactory. Healthier elders more likely to receive support than those with deteriorated health.

							yield more support.					
Ebimgbo et al., Community, Work & Family (2022)	24 elderly individuals (80+) and 16 caregivers (ages 30–46)	To ascertain the extent to which elderly individuals receive support from family and community networks	Qualitative	Focus group discussions and In-depth Interviews	Family ties are gradually disintegrating.	Caregivers predominantly educated; women more likely to prioritize education over caregiving responsibilities.	Families provide greater financial/health support than communities; broader range of social support (financial, material, health, instrumental) yet often inadequate due to competing responsibilities.					Participants recognized the need for non-family members to assist elderly individuals.
Ebimgbo & Okoye, Journal of Population Ageing (2024)	58 left-behind elderly individuals in Abia, Anambra, Ebonyi, Enugu, and Imo	To examine challenges of left-behind older family members of international migrants in south-east Nigeria.	Qualitative	In-depth Interviews and Focus Group Discussions	Migration of younger family members disrupts traditional care and support, leading to neglect.		Left-behind elders resorted to unhealthy coping; loneliness and lack of assistance impeded IADLs, affecting ADLs.					
Eboiyehi & Onwuzuruigbo, Journal of Sociology and Anthropology (2014)	32 IDIs and 8 FGDs among men and women aged 60+	To examine the nature of care and support system for the aged and coping strategies among the Esan of	Qualitative	In-depth Interviews and Focus Group Discussions	Old age in the Esan community is perceived via notions of purity/virtuous living; intergenerational exchanges	Men: subsistence farming, contributions, pension, provider support, menial jobs. Women: petty trading,	Extended family ties diminishing; rural–urban migration and nuclearization have reduced care/support; changes influenced by out-migration,					

		South-South Nigeria			underpin care.	farming, provider support, alms begging.	unemployment, Westernization, industrialization.			
Fajemilehin, Africa Journal of Nursing and Midwifery (year not provided)	150 elderly aged 70+ and their primary support providers in Ife/Ijesa zone, Osun State	To document the experiences of caregiving against socio-cultural and economic change	Mixed methods	Open-ended questionnaires and in-depth interviews	Westernization and education lead to rural–urban migration of children, resulting in lack of care; reliance on maids/house boys diminished.		Collapse of the traditional extended family system reduced closeness and social support; female elderly relatives lived with married children to provide guidance/support.	Westernization/education-driven migration reduced availability of family caregivers.		
Iwuagwu, Ngwu & Ekoh, Journal of Population Ageing (2022)	14 elderly individuals caring for their very old parents (rural SE Nigeria)	To explore the challenges of female elderly individuals caring for very old parents	Qualitative (Descriptive)	Interviews		Caregiving predominantly shouldered by older women contributed to financial hardship.			Psychological burden; inability to speak up increases burden; social burden (marital/sibling conflict); physical health burden.	
Iwuagwu, Ugwu, Ugwuanyi et al., African Journal of Social Work (2022)	195 respondents with caregiving experience in Enugu State	To investigate family caregivers' awareness and perceived access to formal	Quantitative	Questionnaires		Gender not an important factor in predicting access to support services.		Financial constraints, lack of awareness, and lack of social support affect access; place of residence can be barrier/facilitator.	Education level predicts access to formal supports.	Most respondents aware of formal supports; lack of services negatively impacts

		support services for elderly individuals in Enugu State								elders; access challenges persist.
Mayston et al., PLoS One (2017)	60 interviews in 24 households (Peru, Mexico, China & Nigeria)	To explore the social and economic effects of caring for an older dependent person; pathways to economic vulnerability	Mixed methods (Quantitative & Qualitative)	In-depth narrative style interviews		Women were de facto main caregivers in most households; sometimes paid non-family caregivers used.		Severe economic hardship; limiting food/consumption/healthcare due to lack of funds; caregiving and filial duty decreased earning potential and curtailed careers.		Governments were largely uninvolved in care/support of older dependents.
Namadi, Journal of Applied, Management and Social Sciences (2016)	100 family caregivers and 100 elderly recipients in Kano, Nigeria	To examine interaction patterns and perceived care satisfaction among family caregivers and elderly recipients	Mixed methods (Quantitative & Qualitative)	Questionnaires and in-depth interviews		Females experience chronic ailments earlier; daughters-in-law and sons/daughters primary caregivers; females comprise ~60% of caregivers; daughters-in-law report lower satisfaction.	Reciprocity and obligation motivate continuous family care.		Multiple health issues common; caregivers report moderate satisfaction; recipients prefer opportunities for self-care; many caregivers have secondary/primary education.	

Odaman & Ibiezugbe, IFE PsychologIA (2014)	514 respondents in households with elderly persons (65+) in Edo State	To investigate social and economic remittances from relatives to the elderly Edo people	Quantitative	Questionnaires		Elderly females received more food remittances (gender imbalance in assistance).		Socioeconomic remittances poor; urbanization/industrialization/modernization disintegrated extended families; declining economy/unemployment hinder children's support; lack of income increased dependence and health problems; hindered access to healthcare.		Many respondents reported receiving no medical support.
Ojifinni & Uchendu, Pan African Medical Journal (2022)	1,119 adult caregivers aged 18–59 in Oyo State, Nigeria	To assess burden of care experienced by caregivers of elderly persons in family settings	Qualitative study (case study)	Interviews; structured questionnaires		Gender was not associated with burden of care.	Burden not necessarily influenced by quality of prior relationship.		Higher burden in rural vs urban; severe when elderly dependent for ADLs; associated with wealth index, relationship, prior relationship quality, duration; highest among spousal caregivers.	
Okoye, International Journal of Education and Ageing (2012)	530 adults (40+ years, mostly well-educated)	To examine effects of gender, culture, and education in caregiving	Mixed methods	Questionnaires and in-depth interviews		Females received more food remittances.		Socioeconomic remittances poor; urbanization/industrialization/modernization disrupt extended families; economic decline/unemployment hinder support; lack of income affects independence, health, access to healthcare.		Many respondents reported receiving no medical support.
Peil, Journal of	668 men and 336	To explore factors	Quantitative study	Interviews (plus	Rural elderly maintain	Age and gender	Elderly continue exchange			

Comparative Family Studies (1991)	women in three Southern Nigerian cities	affecting support elderly people receive from family members		observation, home visits, discussions)	exchange relationships more extensively than urban parents.	shape type/amount of support: rural men and older urban women receive least; fathers provide material support; mothers provide services; older women more likely to live alone; rural women <75 receive more gifts; very elderly fathers receive better support.	relationships within families.			
Shofoyeke & Amosun, Journal of Social Sciences (2014)	684 principals, head teachers, administrators and planners in	To examine care and support for elderly people in Nigeria	Quantitative (Survey)	Questionnaires		Men generally more aware of nearby elderly than women		Children neglect elders due to poverty from unemployment/underemployment and beliefs (e.g., witchcraft).		Many elderly lack basic necessities (water, decent housing);

	four of six geopolitical zones					(regional variation); no relationship between sex and knowledge of elder residence.			lack of clear welfare, age security, subsidized health services, adequate homes across government levels.
Tanyi et al., Cogent Social Sciences (2018)	3 local government chairpersons in Nsukka LGA, Enugu State (social sciences background; knowledgeable on issues concerning the elderly)	To analyze current policy lacuna and future issues concerning older persons in Nigeria	Qualitative	Interviews and interview narratives	Nigerian communities culturally respect elders; families expected to care for them. No cultural practices preventing government from caring for elders.			Care varies by wealth and well-being of families/communities.	Nigeria lacks a national social security system; faith-based groups and citizens' committees may provide care depending on resources; dysfunctional pensions and collapse of traditional family care impact elders.
Wahab & Adedokun, International Union for	250 respondents (125 per sex) in	To examine changes in family structure	Quantitative	Structured interview aligned with	Reciprocity and obligation were the		Family structure/roles central to care; reciprocity/oblig		Majority preferred institutionalized care for

the Scientific Study of Population (2012)	Ikotun-Igando, Alimosho LGA, Lagos State	and care provision for the elderly in Nigeria		questionnaires	main motivators for continuous family care.		ation motivate care; quality of care declined due to family-structure changes; modernization, industrialization, population growth, urbanization, nuclearization drive changes.					the elderly over traditional care.

Quality assessment of narrative review articles

Supplementary File S4

The Scale for the Assessment of Narrative Review Articles (SANRA).

Author (year)	Appraisal questions						
	Justification for the article's importance for readership	Statement of concrete aims or formulation of questions	Description of the literature search	Referencing	Scientific reasoning	Appropriate presentation of data	Sum score
**Animasahun & Chapman 2022)**	2	0	2	2	1	2	9

Note: 2=High standard, 1=Vague, 0=Low standard.

Quality assessment for qualitative studies

Supplementary File S5

The Mixed Methods Appraisal Tool (MMAT).

Author (year)	Appraisal questions					
	Is the qualitative approach appropriate to answer the research questions?	Are the qualitative data collection methods adequate to address the research questions?	Are the findings adequately derived from the data?	Is interpretation of results sufficiently substantiated by data?	Is there coherence between qualitative data sources, collection and analysis?	Rating
**Akinrolie et al (2020)**	Y	Y	Y	Y	Y	H
**Ebimgbo et al. (2022)**	Y	Y	Y	Y	Y	H
**Ebimgbo & Okoye (2022)**	Y	Y	Y	Y	Y	H
**Ebimgbo et al. (2019)**	Y	Y	Y	Y	Y	H
**Eboiyehi & Onwuzuruigbo (2014)**	Y	Y	Y	Y	Y	H
**Iwuagwu, Ngwu & Ekoh (2022)**	Y	Y	Y	Y	Y	H

**Ojifinni & Uchendu (2022)**	Y	Y	Y	Y	Y	H
**Tanyi et al. (2018)**	Y	Y	Y	Y	Y	H

Note: Y= Yes, N=NO, CT=Can't Tell, H=High quality, L=Low quality.

Quality assessment for mixed method studies.

Supplementary File S6

The Mixed Methods Appraisal Tool (MMAT).

	**Is there an adequate rationale for using a mixed methods design to address the research question?**	**Are the different components of the study effectively integrated to answer the research question?**	**Are the outputs of the integration of qualitative and quantitative components adequately interpreted?**	**Are divergencies and inconsistencies between quantitative and qualitative results addressed?**	**Do the different components of the study adhere to the quality criteria of each tradition of the method involved?**	
						Rating
**Ani & Isiugo-Abanihe (2017)**	Y	Y	Y	Y	Y	H
**Fajemilehin 2000)**	N	CT	Y	N	Y	L
**Mayston et al. PLOS ONE (2017)**	CT	Y	Y	Y	Y	H
**Namadi (2016)**	Y	Y	Y	Y	Y	H
**Okoye (2012)**	N	Y	Y	N	Y	H

Note: Y= Yes, N=NO, CT=Can't Tell, H=High quality, L=Low quality.

Quality assessment for quantitative studies

Supplementary File S7

The Mixed Methods Appraisal Tool (MMAT).

**Author/Year**	**Is the sampling strategy relevant to address the research?**	**Is the sample representative of the target population?**	**Are measurements appropriate?**	**Is the risk of non-bias low?**	**Is the statistical analysis appropriate to answer the research**	
						Rating
**Ebimgbo and Okoye**	CT	Y	Y	Y	Y	H

**(2017)**						
**Iwuagwu, Ugwu, Ugwuanyi et al. (2022)**	Y	Y	Y	CT	Y	H
**Odaman & Ibiezugbe (2014)**	Y	Y	Y	CT	Y	H
**Peil (1991)**	**N**	**N**	**CT**	**N**	**Y**	**L**
**Shofoyeke & Amosun (2014)**	Y	Y	Y	Y	Y	H
**Wahab & Adedokun (2012)**	Y	Y	CT	CT	Y	H

Note: Y= Yes, N=NO, CT=Can't Tell, H=High quality, L=Low quality.
